# Factors associated with recurrent malaria episodes among children under five at Kayunga Regional Referral Hospital in Kayunga District, Central Uganda

**DOI:** 10.1371/journal.pone.0320112

**Published:** 2025-06-12

**Authors:** Derick Modi, Marvin Musinguzi, Patricia Pita, Eustes Kigongo, Amir Kabunga, Julius Kayizzi, Deo Kasaija, Voni Alice Khanakwa, Oscar Simon Alyao, Julius Lubangakene, Tom Murungi, Christopher Okullo Oneka, Marc Sam Opollo

**Affiliations:** 1 Department of Community Health, Faculty of Public Health, Lira University, Lira City, Uganda; 2 Department of Midwifery, Faculty of Nursing and Midwifery, Lira University, Lira City, Uganda; 3 Department of Environmental Health, Faculty of Public Health, Lira University, Lira City, Uganda; 4 Department of Psychiatry, Faculty of Medicine, Lira University, Lira City, Uganda; University of Ghana College of Health Sciences, GHANA

## Abstract

**Background:**

Malaria poses a substantial global challenge and continues to be a major cause of mortality and morbidity in numerous developing nations. Children under the age of five in low- and middle-income countries such as Uganda are the most affected. However, there remains a deficiency in knowledge regarding recurrent malaria episodes in Uganda. We determined the prevalence and factors associated with recurrent malaria episodes among children under five at Kayunga Regional Referral Hospital.

**Methodology:**

This was a cross-sectional study conducted among children under five at Kayunga Regional Referral Hospital in central Uganda. The data was collected among 250 consecutively sampled participants who were caring for children under five. Data was collected using a researcher-administered questionnaire and analyzed at univariate, bivariate, and multivariate levels.

**Results:**

A total of 250 participants participated in the study with a response rate of 98.45%. The prevalence of recurrent malaria episodes was 84% (210). The factors significantly associated with recurrent malaria episodes were; children from houses that were annually sprayed (aOR; 8.93, 95%CI,2.11–37.81), children from houses that were not sprayed (aOR; 3.80,95%CI,1.27–9.41, p = 0.017), children who were treated with quinine antimalarial in the previous infection (aOR, 0.28, 95%CI,0.12–0.65) and children who were residing in a house whose windows were closed at 7–8 pm (aOR, 8.31, 95%CI, 2.21–10.27).

**Conclusion:**

The recurrence of malaria episodes among children under five is significantly high, suggesting the possibility of malaria resistance. Importantly, quinine remains a robust alternative treatment for complicated malaria, owing to its significant efficacy against malaria parasites in regions of moderate to high transmission rates. Malaria prevention programs should consider biannual indoor residual spraying in high malaria transmission areas using vector-susceptible insecticides.

## Background

Malaria remains one of the leading public health burdens in the world despite the remarkable achievements made towards its control and prevention since the beginning of the second millennium [1]. Globally, according to the World Malaria Report 2020, there were an estimated 241 million cases of malaria and 627,000 malaria deaths [2]. In Africa, four African countries accounted for just over half of all malaria deaths worldwide: Nigeria (31.9%), the Democratic Republic of the Congo (13.2%), the United Republic of Tanzania (4.1%), and Mozambique at 3.8% [2]. Uganda has the 3^rd^ highest proportion of malaria cases (5%) and the 8^th^ highest level of deaths (3%) [3]. It also has the highest proportion of malaria cases in East and Southern Africa, 23.7% among children under five [4]. According to the WHO, children under the age of 5 years are the most affected, with more than two-thirds of deaths due to malaria [5]. The recurrent malaria episodes in children under five present a higher risk of mortality because children under five have an underdeveloped immune system compared to adults. This makes them less capable of fighting off infections like malaria. Recurrent infections can further weaken their immune responses, leading to higher vulnerability and risk of severe complications [6].

A recurrent malaria infection is a newly detectable episode of blood-stage parasitemia occurring after a previous infection [7]. This can be due to relapse of P. vivax and P. ovale infections [2], usually occurring after 48–72 hours when the parasites come out of hibernation and begin invading red blood cells [7]. The prevalence of recurrent malaria infection can vary depending on various factors such as the geographical location, intensity of malaria transmission, access to healthcare, and the effectiveness of malaria control interventions [8]. Recurrent episodes of malaria among children under five can contribute to poor nutrition, weight loss, and stunted growth [9]. Malnutrition diminishes a child’s immune system, rendering them more vulnerable to subsequent infections [10]. On the other hand, emotional and psychological consequences include Chronic illness, such as recurrent episodes of malaria, cognitive impairment, among others, which can harm a child’s well-being [11].

Uganda has the sixth highest number of annual deaths from malaria in Africa, as well as some of the highest reported malaria transmission rates in the world, with approximately 16 million cases reported in 2013 and over 10,500 deaths annually. In addition, malaria has an indirect impact on the economy and development in general [12]. However, clinically diagnosed malaria is the leading cause of morbidity and mortality, accounting for 30–50% of outpatient visits at health facilities, 15–20% of all hospital admissions, and up to 20% of all hospital deaths [13]. The government of Uganda has made efforts to provide LLITN to households and IRS to control malaria infections in children under five [14].

Despite extensive research and interventions, recurrent malaria episodes persist, exacerbating child morbidity and mortality. Kayunga has a malaria prevalence of 18.4% according to the Malaria Indicator Survey 2020 [15]. At Kayunga Regional Referral Hospital, the factors contributing to these recurrent episodes are not well understood, highlighting a critical gap in the literature. Existing studies primarily focus on initial malaria incidence and general prevention measures [16–18], with limited attention to the specific determinants of recurrence in this vulnerable age group. Factors such as socio-economic conditions, healthcare access, and specific local epidemiological characteristics have not been comprehensively examined in the context of Kayunga. Addressing these gaps is crucial for developing targeted strategies to reduce the burden of recurrent malaria and improve health outcomes for children under five in this region. We thus determined the prevalence and factors associated with recurrent malaria episodes among children under five at Kayunga Regional Referral Hospital, Kayunga district in central Uganda.

## Materials and methods

### Study design and setting

We employed a cross-sectional study design that used quantitative methods to collect and analyze the data. The study was conducted from 10th to 25th March 2024. The study was conducted at Kayunga Regional Referral Hospital’s Outpatient Department and the pediatric ward. Kayunga district is located in Central Uganda and has a grid reference of 0° 59 9.6648” N and 32° 51 12.8736” E. It has an elevation of 1063m (3488 ft). Kayunga District is 74 Km East of Kampala City, bordered by Mukono District to the south, Jinja and Buikwe to the east, Kamuli to the northeast, Amolator & Apac in the North, Luwero in the west and Nakasongola to the northwest.

Kayunga district has a malaria prevalence of 18.4%, according to the Malaria Indicator Survey 2020 [15], although little is known about recurrent malaria episodes among children under five.

### Study population

The study population consisted of caretakers, parents, or guardians of children under five who came to access care at Kayunga Regional Referral Hospital. We recruited caretakers, parents, or guardians of children under five who had consented and were available at the pediatric ward or outpatient clinic of Kayunga Regional Referral Hospital at the time of data collection. We excluded caretakers who had not spent at least one month with the children before data collection.

### Sample size and sampling technique

The sample size was determined using the Kish Leslie (1965) formula (n = Z^2^PQ/d^2^) [19]. Z = 1.96%, d = 0.05, p = 18.4%, which is the malaria prevalence among children under five according to the Uganda malaria indicator survey. This resulted in a sample size of 231. When adjusted for a non-response rate of 10%, an overall sample size of 254 participants was generated.

A consecutive sampling method was used to select participants for data collection. The study included all caregivers of children under the age of five who met the inclusion criteria and sought health care at Kayunga Regional Referral Hospital’s Outpatient Department (OPD) and the pediatric ward.

### Data collection tools

Data was collected using a researcher-administered questionnaire. The questionnaire was adapted from Simões and colleagues and tailored to the study setting [20]. Questions were divided into three sections, including socio-demographics (age, religion, economic status, etc.), household factors (Poor housing construction, lack of mosquito control, poor sanitation and hygiene, stagnant water, lack of knowledge and awareness), and practices (use of insecticide-treated bed nets, indoor residual spraying, larval source management, self-medication, over using antimalarial). Before the actual data collection, the tool was also pretested at Lira University Teaching Hospital and translated to Luganda, the local language spoken by the majority of residents at Kayunga Regional Referral Hospital.

We defined recurrent malaria episodes as having suffered from at least two or more episodes of malaria infection within 30 days, with at least one episode being diagnosed using a rapid diagnostic test (RDT) or microscopy [21]. Thus, it was measured using a binary response (yes/no), where those whose children suffered from at least two or more episodes of malaria infection within 30 days, with at least one episode being diagnosed using a rapid diagnostic test (RDT) or microscopy, were categorized as “yes”. On the other hand, those whose children had not suffered at least two malaria episodes or those whose malaria was not medically tested and diagnosed were categorized as “no”.

### Data collection procedure

Upon ethical clearance from the Lira University Research Ethics Committee, the researcher sought permission from the District Health Officer, the principal medical officer, and other relevant authorities at Kayunga Regional Referral Hospital to conduct the study. The researcher then trained five research assistants to collect data for the participants who met the eligibility criteria, and data collection was done by conducting researcher-administered interviews using semi-structured questionnaires after getting consent from the participants. Before the data collection process, written informed consent to participate in the study was sought from the participants. Recurrent malaria infection was self-reported by the caretakers of the children and was validated by asking the caretakers to provide at least one record of a previous malaria diagnosis from a hospital or clinic.

### Data management and analysis

Data was entered, cleaned, and analyzed in SPSS version 26. During univariate analysis, descriptive statistics were used to summarize the data and determine the prevalence of recurrent malaria episodes, including frequencies and percentages. At bivariate analysis, a bivariate logistic regression was performed between the independent variables and the dependent variable at a 95% confidence interval. Crude odds ratios (COR) were used as measures of association. Variables with P ≤ 0.05 were considered to have significant associations with the dependent variable. In multivariate analysis, variables with P ≤ 0.2 at the bivariate level were included in the multivariate logistic regression at a 95% confidence interval. Adjusted Odds Ratios were used to measure association. Variables that had P ≤ 0.05 at multivariate logistic regression were considered to be significantly associated with the dependent variable.

### Ethical considerations

Ethical approval to conduct the study was obtained from the Lira University Research Ethics Committee (LUREC-2023-66). Administrative clearance to conduct the study was sought from the principal medical officer and other relevant authorities at Kayunga Regional Referral Hospital. Written informed consent was sought from all the participants in this study, who were mothers and guardians of the children, after sharing with them the study’s objectives and possible benefits and risks. Identifiers such as names, phone numbers, or personal addresses were not included in the questionnaires to protect the participants’ privacy. Codes were used to identify participants. The hard copy of the questionnaires was kept under lock and key and was only accessible by the research team.

## Results

Out of the 254 participants in the study, data were only collected from 250 participants at Kayunga Regional Referral Hospital in Central Uganda. This resulted in a response rate of 98.45%.

### Recurrent malaria episodes and socio-demographic characteristics of the respondents

The following socio-demographic variables were significantly associated with recurrent malaria episodes. Children who were not in school experienced many malaria episodes (cOR; 0.50, 95%CI, 0.252–0.976, P = 0.04), children whose parents had a monthly income of 330,000–930,000ugx, were more likely to experience recurrent malaria episodes (cOR; 2.83, 95%CI, 1.098–7.287, p = 0.03), children who had underlying conditions had many recurrent episodes of malaria (cOR; 2.69, 95%CI, 1.30–5.55, p = 0.01), children who had other underlying conditions had more malaria episodes (cOR; 2.31, 95%CI, 0.50–10.65, P = 0.01) (Table 1).

**Table 1:** Showing the bivariate analysis results of the socio-demographic characteristics associated with recurrent malaria episodes among children under five at Kayunga Regional Referral Hospital.

Variable	Frequency N (%)	Malaria recurrence		cOR (95% CI)	P value
		No recurrence	Recurrence		
Malaria recurrent episodes		16%	84%		
**Age of the guardian**					
15-34	3 (1.20)	1 (33.30)	2 (66.70)	Ref	
35-54	128 (51.20)	21 (16.40)	107 (83.60)	0.57 (0.41-8.05)	0.681
55-74	101 (40.40)	15 (14.90)	86 (85.10)	1.46 (0.44-4.86)	0.542
75-above	18 (7.20)	4 (22.20)	14 (77.80)	1.64 (0.47-5.66)	0.440
**Age of the child in months**					
3-12, infant	61 (24.40)	12 (19.70)	49 (80.30)	Ref	
13-48, toddler	162 (64.80)	24 (14.80)	138 (85.20)	0.93 (0.29-2.96)	0.900
49-above, children.	27 (10.80)	5 (18.50)	22 (81.50)	1.31 (0.45-3.79)	0.621
**Gender**					
Male	115 (46.00)	20 (17.40)	95 (82.60)	Ref	
Female	135 (54.00)	21 (15.60)	114 (84.40)	0.88 (0.45-1.71)	0.701
**Marital status**					
Married	132 (52.80)	20 (15.20)	112 (84.80)	Ref	
Cohabiting	49 (19.60)	8 (16.30)	41 (83.70)	1.30 (0.60-2.80)	0.501
Single	69 (27.60)	13 (18.80)	56 (81.20)	1.19 (0.45-3.13)	0.732
**Religion**					
Catholics	55 (22.00)	10 (18.20)	45 (81.80)	Ref	
Protestant	91 (36.40)	19 (20.90)	72 (79.10)	0.49 (0.14-1.68)	0.251
Muslim	63 (25.20)	8 (12.70)	55 (87.30)	0.41 (0.13-1.29)	0.132
Others	41 (16.40)	4 (9.80)	37 (90.20)	0.74 (0.21-2.65)	0.651
**Education level**					
Primary	83 (33.20)	14 (16.90)	69 (83.10)	Ref	
Secondary	84 (33.60)	16 (19.00)	68 (81.00)	0.67 (0.22-1.99)	0.472
Tertiary	41 (16.40)	6 (14.60)	35 (85.40)	0.574 (0.20-1.69)	0.321
None	42 (16.80)	5 (11.90)	37 (88.10)	0.79 (0.22-2.82)	0.710
**Schooling status**					
In school	87 (34.8)	20 (23.0)	67 (77.0)	Ref	
Not in school	163 (65.2)	21 (12.9)	142 (87.1)	0.495 (0.252-0.976)	0.040*
**Monthly household income ($)**					
20-84	158 (63.20)	18 (11.40)	140 (88.60)	Ref	
85-244	62 (24.80)	15 (24.20)	47 (75.80)	2.83 (1.10-7.29)	0.030*
245-above	30 (12.00)	8 (26.70)	22 (73.30)	1.14 (0.42-3.09)	0.801
**Presence of any underlying condition**					
Yes	122 (48.80)	12 (9.80)	110 (90.20)	Ref	
No	128 (51.20)	29 (22.70)	99 (77.30)	2.69 (1.30-5.55)	0.010*
**Type of Underlying Condition**					
Sickle cell	12 (4.80)	3 (25.00)	9 (75.00)	Ref	
Measles	24 (9.60)	4 (16.70)	20 (83.30)	0.87 (0.22-3.42)	0.871
Chickenpox	18 (7.20)	2 (11.10)	16 (88.90)	1.44 (0.46-4.57)	0.531
Others	71 (28.40)	4 (5.60)	67 (94.40)	2.31 (0.50-10.65)	0.010*
No condition.	125 (50.00)	28 (22.40)	97 (77.60)	4.84 (1.62-14.42)	0.281
**Place of residence**					
Rural	163 (65.20)	24 (14.70)	139 (85.30)	Ref	
Urban	87 (34.80)	17 (19.50)	70 (80.50)	1.41 (0.71-2.79)	0.331

*Level of significance at p < 0.05, ***level of significance at p < 0.001, p ≤ 0.05.

### Practices towards the prevention of malaria

At bivariate analysis, children in the houses which were spayed annually were less likely to experience recurrent malaria episodes (cOR;0.23,95%CI,0.09–0.58, P = 0.00), children who were practicing self-medication were more likely to experience recurrent malaria episodes (cOR2.25,95%CI,2.25–1.13, P = 0.02), children who were using artesunate antimalaria drug were less likely to experience recurrent malaria episodes (cOR;0.23,95%CI,0.00–0.02,0.01), children staying in houses where windows were closed from 7–8 pm had more recurrent malaria episodes (cOR;2.19,95%CI,1.10–4.36, P = 0.03) (Table 2).

**Table 2:** Showing the bivariate analysis of the practices towards prevention of malaria with recurrent malaria episodes among children under five at Kayunga Regional Referral Hospital.

Variable	Frequency, N (%)	Malaria recurrence		cOR (95%CI)	P value
		No recurrence	Recurrence		
**Spraying the house with insecticide**					
Yes	81 (32.40)	17 (21.00)	64 (79.00)	Ref	
No	169 (67.60)	24 (14.20)	145 (85.80)	0.62 (0.31-1.24)	0.180
**Period sprayed**					
Monthly	24 (9.60)	10 (45.50)	12 (54.50)	Ref	
Annually	50 (20.00)	4 (8.00)	46 (92.00)	0.23 (0.09-0.58)	<0.001***
Others	7 (2.80)	3 (42.90)	4 (57.10)	1.91 (0.63-5.77)	0.260
I don’t spray	169 (67.60)	24 (14.20)	145 (85.80)	0.221 (0.05-1.05)	0.060
**Use of treated mosquito net**					
Yes	212 (84.80)	35 (16.50)	177 (83.50)	Ref	
No	38 (15.20)	6 (15.80)	32 (84.20)	0.95 (0.37-2.44)	0.950
**Self-medicate the child**					
Yes	133 (15.20)	15 (11.30)	118 (88.70)	Ref	
No	117 (46.80)	26 (22.20)	91 (77.80)	2.25 (2.25-1.13)	0.020*
**Antimalarials commonly used to treat malaria**					
Coartem	137 (57.20)	15 (10.90)	122 (89.10)	Ref	
Quinine	64 (25.60)	20 (31.30)	44 (68.80)	0.86 (0.18-4.04)	0.851
Artesunate	28 (11.60)	4 (14.30)	24 (85.70)	0.23 (0.00-0.02)	0.011*
p-alaxin	21 (4.40)	2 (9.50)	19 (90.50)	0.63 (0.10-3.82)	0.610
**Always use the recommended anti-malarial**					
Yes	169 (67.60)	27 (16.00)	142 (84.00)	Ref	
No	81 (32.40)	14 (17.30)	67 (82.70)	1.09 (0.54-2.23)	0.791
**Use of mosquito repellents**					
Yes	73 (29.20)	15 (20.50)	58 (79.50)	Ref	
No	177 (70.80)	26 (14.70)	151 (85.30)	0.66 (0.33-1.35)	0.260
**At what time do you close windows every day**					
3-4 pm	5 (2.00)	1 (20.00)	4 (80.00)	Ref	
5-6 pm	166 (66.40)	21 (12.70)	145 (87.30)	1.27 (0.13-12.03)	0.840
7-8 pm	79 (31.60)	19 (24.10)	60 (75.90)	2.19 (1.10-4.36)	0.031*
**Time to be indoors**					
6-7 pm	82 (32.80)	17 (20.70)	65 (79.30)	Ref	
8-9 pm	125 (50.00)	19 (15.20)	106 (84.80)	0.50 (0.17-1.47)	0.211
10-11 pm	43 (17.20)	5 (11.60)	38 (88.40)	0.734 (0.26-2.10)	0.570

*Level of significance at p < 0.05, ***level of significance at p < 0.001, p ≤ 0.05.

### Household practices

At bivariate analysis, the children whose homes were near the bush were more likely to experience recurrent malaria episodes (cOR; 2.36, 95% CI,1.18–4.72, P = 0.02) (**Table 3**).

**Table 3:** Household practices associated with recurrent malaria episodes among children under five at Kayunga Regional Referral Hospital.

Variable	Frequency N (%)	Malaria recurrence		cOR (95%CI)	P value
		No recurrence	Recurrence		
**Home Near a Bush**					
Yes	180 (72.00)	23 (12.80)	157 (87.20)	Ref	
No	70 (28.00)	18 (25.70)	52 (74.30)	2.36 (1.18-4.72)	0.020*
**Household near a water body**					
Yes	116 (46.40)	21 (18.10)	95 (81.90)	Ref	
No	134 (53.60)	20 (14.90)	114 (85.10)	`0.80 (0.41-1.55)	0.501
**Household Number**					
3-8	178 (71.20)	31 (17.40)	147 (82.60)	Ref	
9-14	58 (23.20)	7 (12.10)	51 (87.90)	1.29 (0.34-4.91)	0.710
15-above	14 (5.60)	3 (21.40)	11 (78.60)	1.99 (0.44-8.92)	0.371
**Total number of children in the home**					
1-3	53 (21.20)	8 (15.10)	45 (84.90)	Ref	
4-6	150 (60.00)	27 (17.90)	124 (82.10)	0.84 (0.27-2.64)	0.770
7-above	46 (18.40)	6 (13.00)	40 (87.00)	0.69 (0.27-1.79)	0.440
**Availability of stagnant water in the home**					
Yes	67 (26.90)	11 (16.40)	56 (83.60)	Ref	
No	183 (73.20)	30 (16.40)	153 (83.60)	0.99 (0.47-2.13)	0.991
**Households experiencing flooding**					
Yes	183 (73.20)	29 (15.80)	154 (84.20)	Ref	
No	67 (26.80)	12 (17.90)	55 (82.10)	1.16 (0.55-2.43)	0.701

*Level of significance at p < 0.05, ***level of significance at p < 0.001, p ≤ 0.05.

### Factors associated with the prevalence of recurrent malaria episodes among children under five at Kayunga Regional Referral Hospital

At Multivariate analysis, children from houses that were annually sprayed (aOR; 8.93,95% CI,2.11–37.81, p = 0.003), as well as those from households that didn’t spray at all (aOR; 3.80, 95% CI,1.27–9.41, p = 0.017), were more likely to experience recurrent malaria episodes. Additionally, children who were using quinine antimalarial were less likely to experience recurrent malaria episodes (aOR, 0.28,95%CI,0.12–0.65, P = 0.003), children who were residing in a house whose windows were closed at 7–8 pm were more likely to experience recurrent malaria episodes (aOR, 8.31,95%CI, 2.21–10.27 P = 0.002) (Table 4).

**Table 4:** Shows the factors associated with recurrent malaria episodes among children under five at Kayunga Regional Referral Hospital.

Variable	cOR	P value	aOR	P value
**Period sprayed**				
Monthly	Ref		Ref	
Annually	0.23 (0.09-0.58)	<0.001***	8.93 (2.11-10.81)	0.003*
Others	1.91 (0.63-5.77)	0.26	0.78 (0.12-5.20)	0.812
I don’t spray	0.22 (0.05-1.05)	0.06	3.80 (1.27-9.41)	0.017*
**Commonly used antimalarials to treat malaria**				
Coartem	Ref		Ref	
Quinine	0.86 (0.18-4.04)	0.85	0.28 (0.12-0.65)	0.003*
Artesunate	0.23 (0.00-0.02)	0.011*	0.61 (0.17-2.19)	0.446
p-alaxin	0.63 (0.10-3.82)	0.61	2.22 (0.39-8.65)	0.366
**At what time do you close windows every day**				
3-4 pm	Ref		Ref	
5-6 pm	1.27 (0.13-12.03)	0.84	2.11 (0.36-12.96)	0.721
7-8 pm	2.19 (1.10-4.36)	0.031*	8.31 (2.21-10.27)	0.002*

*Level of significance at p < 0.05, ***level of significance at p < 0.001, p ≤ 0.05.

## Discussion

Our results showed an 84% prevalence of recurrent malaria episodes in children under five, a notably high figure compared to other studies. For instance, a systematic review reported a 30% prevalence of malaria recurrences within 28 days after the initial treatment [22]. Similarly, a study conducted in Western Thailand found that 9.78% of individuals were infected with malaria two or more times in 2019 [7]. The lower prevalence of recurrent malaria in these studies can be attributed to adherence to preventive practices such as indoor residual spraying, avoiding self-medication (which can lead to antimalarial drug resistance), and preventing childhood illnesses like measles and chickenpox, which weaken children’s immunity and make them more vulnerable to malaria. These measures collectively reduce malaria recurrence risk by addressing environmental factors and individual susceptibility.

Our findings indicate that children from households that were annually sprayed or from households that didn’t spray at all were more likely to experience recurrent malaria episodes. This could be explained by the fact that the sprayed vector population could have been unsusceptible to the insecticides applied. This finding contrasts with a study conducted in Northern Uganda, which found that indoor residual spraying reduced the chances of recurrent malaria infections in children under five [23].In addition, findings from Eastern Uganda revealed that indoor residual spraying and insecticide-treated mosquito nets in homes provided additional protection against mosquito bites and consequently reduced malaria incidence [24]. The discrepancy between these studies could be attributed to differences in the study settings and other behaviours related to malaria prevention. WHO recommends universal LLIN coverage as the cornerstone of vector control, adding IRS with a non-pyrethroid insecticide only where confirmed pyrethroid resistance and persistently high malaria transmission undermine LLIN efficacy [25]. In Kayunga district, where malaria incidence remains high [26] despite >90% LLIN use among households [27], combining IRS (using a non-pyrethroid active ingredient) and LLINs is warranted to prevent further resistance and reduce transmission [28]. In addition, the malaria prevention programs should consider biannual indoor residual spraying in high malaria transmission areas using vector-susceptible insecticides.

Our results indicate that children treated with quinine in a previous malaria infection were less likely to experience recurrent malaria episodes than those who used other anti-malarial medications. This could be possibly due to quinine being always carefully prescribed and administered with close monitoring to hospitalized children with complicated malaria, which could lead to complete eradication of the parasites. This finding is consistent with a study conducted in Indonesia, which showed that the use of quinine reduces the chances of malaria infection recurrence [29]. This efficacy is likely due to quinine’s continued effectiveness against drug-resistant Plasmodium falciparum strains, ensuring successful treatment [30]. These results underscore the importance of continuing to use quinine as an alternative treatment for malaria, particularly in areas where drug resistance is prevalent. The findings also imply that complicated malaria and poorly managed malaria can effectively be managed with quinine in cases where artemisinin-based combination therapy (ACTs) have been initially used.

Our results showed that children who lived in houses with windows closed as late as 7 p.m. - 8 p.m. were more likely to have recurring malaria episodes compared to those who had windows closed earlier. This finding shows the critical importance of the timing of window closure in malaria prevention. Similar studies have indicated that the practice of closing doors and windows is significantly associated with malaria transmission [31,32]. Anopheles mosquitoes are most active during dusk and early evening hours, so closing windows later in the evening exposes children to mosquito bites at a critical time, increasing the likelihood of malaria transmission [33,34]. This extended exposure period can result in more frequent malaria episodes. In Uganda, especially during hot nights in the dry season, people might delay closing windows for ventilation, inadvertently increasing malaria risk [31]. This study highlights the need for community education on the importance of closing windows before dusk as a simple yet effective measure to prevent malaria.

### Study limitations

Our study had some limitations that could have affected the robustness and generalizability of our results. This study was hospital-based and used a cross-sectional design, which could have caused selection bias and difficulty in ascertaining the causal relationship between the independent factors and recurrent malaria among children. This was, however, mitigated by performing multivariate analysis using binary logistic regression to control for possible confounding variables. In addition, our findings could have been affected by recall bias, whereby caretakers and guardians of these children could have forgotten the child’s medical history. However, caretakers were asked to provide at least one record of a previous malaria diagnosis from a hospital or clinic to minimize bias.

## Conclusion

The recurrence of malaria episodes among children under five is significantly high, suggesting the possibility of malaria resistance. Importantly, quinine remains a robust alternative treatment for complicated malaria, owing to its significant efficacy against malaria parasites in regions of moderate to high transmission rates. Malaria prevention programs should also consider biannual indoor residual spraying in high malaria transmission areas using vector-susceptible insecticides and in areas where Long-Lasting Insecticidal Nets (LLIN) are not used.

## Supporting information

S1 File Questionnaire recurrent malaria. (DOCX)

S2 File Recurrent malaria data. (XLSX)
