# Supplementary material for: Factors associated with recurrent malaria episodes among children under five at Kayunga Regional Referral Hospital in Kayunga District, Central Uganda
Source: PLoS One. 2025 Jun 12;20(6):e0320112. doi: 10.1371/journal.pone.0320112 (PMC12161538; doi:10.1371/journal.pone.0320112)
Supplement: S1 File — (DOCX) [file pone.0320112.s001.docx]

|  | **CODE** | **Questionnaire number** |  | | | |
| --- | --- | --- | --- | --- | --- | --- |
|  | **A** | **SECTION A: SOCIO DEMOGRATIC** |  |  |  |  |
| 1 | A1 | What is your Age? | ………………………… | | | |
|  |  | What is the age of the child in months | ………………………… | | | |
| 2 | A2 | What your gender | 1. Male 2. Female | | | |
| 3 | A3 | What is your marital status? | 1. Married 2. Cohabiting 3. Single | | | |
| 4 | A4 | What is your Religion? | 1. Catholic 2. Protestant 3. Muslim 4. Others | | | |
| 5 | A5 | What is your level of education? | 1. Primary 2. Secondary 3. Tertiary 4. None | | | |
| 6 | A6 | Place of residence? | 1. Rural 2. Urban | | | |
|  |  | Is the child in school | 1. Yes 2. No | | | |
|  | A6 | Average house hold income per month | …………………… | | | |
|  | A7 | Does the child have any underlying condition (sick) | 1. YES 2. NO | | | |
|  | A8 | Which kind of under lying condition is that | 1. Sickle cell 2. Measles 3. Chicken pox 4. Others | | | |
|  | **B** | **SECTION B; PERIOD PREVALENCE** |  | | | |
| 1 | B1 | Has the child ever gotten infected with malaria | 1. Yes 2. No | | | |
| 2 | B2 | Does the child have malaria currently | 1. Yes 2. No | | | |
| 3 | B3 | Does the child have symptoms of malaria currently | 1. Yes 2. No | | | |
| 4 | B4 | Estimate the number of times the child has gotten infected with malaria in the previous year | 1. Once 2. Two times 3. Three times 4. Others | | | |
| 5 | B5 | Kindly identify the months the child was diagnosed with malaria in the previous year. (tick the moths) | Jan |  | July |  |
|  |  |  | Feb |  | Aug |  |
|  |  |  | March |  | Sep |  |
|  |  |  | April |  | Oct |  |
|  |  |  | May |  | Nov |  |
|  |  |  | June |  | Dec |  |
|  | C | **SECTION C; PRACTICES TOWARDS PREVENTION OF MALARIA** |  | | | |
| 1 | C1 | Do you spray your house with insecticides? | 1. Yes 2. No | | | |
| 2 | C2 | How often do you spray your house? | 1. Weekly 2. Monthly 3. Annually 4. Others | | | |
| 3 | C3 | Do you use treated mosquito nets | 1. Yes 2. No | | | |
| 4 | C4 | Do you treat the children yourself before visiting a doctor when they show signs and symptoms of malaria | 1. Yes 2. No | | | |
| 5 | C5 | Which anti-malarial are you familiar with to treat malaria | …………………… | | | |
| 6 | C6 | Have you been using that anti malaria each time the child gets infected or when you suspect they are infected? | 1. Yes 2. No | | | |
| 7 | C7 | Do you use mosquito repellants to prevent mosquito bites | 1. Yes 2. No | | | |
| 8 | C8 | At what time do you close windows every day | ……………………. | | | |
| 9 | C9 | At what time are children encouraged to be in doors at home | ……………………. | | | |
|  | **D** | **House hold factors** |  | | | |
| 1 | D1 | Is your house hold near a bush of forest | 1. Yes 2. No | | | |
| 2 | D2 | Is your house hold near a water body | 1. Yes 2. No | | | |
| 3 | D3 | What is the total number of people at your home | **………………………** | | | |
| 4 | D4 | What is the total number of children in the home | **……………………...** | | | |
| 5 | D5 | Is the home having stagnant water all over | 1. Yes 2. No | | | |
| 6 | D6 | Does the home flood in the wet season | 1. Yes 2. No | | | |
